# Cushing’s syndrome in a young woman due to prolonged betamethasone nasal drop use: a case report

**DOI:** 10.1186/s13256-025-05428-3

**Published:** 2025-07-25

**Authors:** Mohammadsadra Shamohammadi, Delaram Eskandari, Amir Ziaee, Seyed Hossein Samadanifard, Haleh Chehrehgosha, Amir Hossein Ghanooni

**Affiliations:** 1 Gastrointestinal and Liver Diseases Research Center, Iran University of Medical Sciences, Tehran, Iran; 2 M.D., Endocrinologist Assistant Professor of Internal Medicine Assistant Professor of Internal Medicine, Iran University of Medical Sciences at Rasool Akram General Hospital, Tehran, Iran; 3 Professor of Endocrinology Department of Endocrinology, Rasool Akram Medical Complex, School of Medicine, Iran University of Medical Sciences, Tehran, Iran; 4 Assistant Professor of Endocrinology & Metabolism Department of Internal Medicine, School of Medicine Hazrat-e Rasool General Hospital Iran University of Medical Sciences Medical Doctor at Iran University of Medical Sciences, Tehran, Iran; 5 Assistant Professor of Endocrinology & Metabolism Department of Internal Medicine, School of Medicine, Iran University of Medical Sciences, Tehran, Iran; 6 M.D., Endocrinologist Assistant Professor of Internal Medicine Assistant Professor of Internal Medicine, Iran University of Medical Sciences at Rasool Akram General Hospital, Tehran, Iran

**Keywords:** Cushing’s syndrome, Betamethasone, Intranasal corticosteroids, Iatrogenic, Case report

## Abstract

**Background:**

Cushing’s syndrome is an uncommon but serious condition caused by long-term exposure to elevated cortisol levels, which is usually iatrogenic in origin. Although systemic corticosteroids are the most frequent agents, the association of intranasal corticosteroids with this condition is remarkably rare.

**Case presentation:**

This report is about a 21-year-old Iranian woman using betamethasone nasal drops for nasal obstruction. The patient presented with weight gain, Amenorrhea, mood disturbances, red purplish striae, and mild hirsutism. Hormonal assessments revealed suppression of the hypothalamic–pituitary–adrenal axis.

**Conclusion:**

This case demonstrates the underappreciated systemic effects of intranasal betamethasone to induce Cushing’s syndrome. It serves as a pivotal reminder of the need for vigilance in prescribing practices and reinforces the importance of early diagnosis to ensure favorable patient outcomes.

## Background

Iatrogenic Cushing’s syndrome (CS) is an endocrine disease caused by long-term or high-dose glucocorticoid use [1]. Although iatrogenic cases are commonly associated with oral or injectable glucocorticoids [2], few reports described CS after the use of intranasal steroid sprays (INS) such as betamethasone in adults [3–7]. Currently, INS is widely used for managing conditions such as allergic rhinitis, nasal polyposis, and other upper airway disorders owing to their localized effects and limited systemic absorption [8, 9]. However, prolonged use, high doses, or using potent formulations can lead to significant systemic absorption, resulting in Hypothalamic–pituitary–adrenal (HPA) axis suppression, and frank CS [10]. Betamethasone nasal spray, a cornerstone in the treatment of nasal congestion, has the potential for systemic absorption by the nasal mucosa, particularly with prolonged or excessive use [11].

This report presents the case of a young woman who developed CS following the overuse of betamethasone nasal drops. It also highlights the importance of detailed patient histories when diagnosing CS and highlights the critical need to educate patients on the proper use and potential risks of steroid therapies to prevent complications. This case report adheres to the case report (CARE) guidelines [12].

### Case presentation

This is the case of a 21-year-old Iranian female who presented with a history of rapid weight gain (30 kg in 8 months), irregular menstrual cycles, and significant mood changes. Her body mass index (BMI) was calculated at 40.07 kg/m^2^, classifying her as obese, and her blood pressure was recorded at 115/75 mmHg. In addition, she exhibited red–purple striae on her abdomen and limbs and mild hirsutism (modified Ferriman–Gallwey Score (FGS) score = 10), prompting admission for further evaluation after multiple outpatient visits yielded no definitive diagnosis. 

**Fig. 1:**
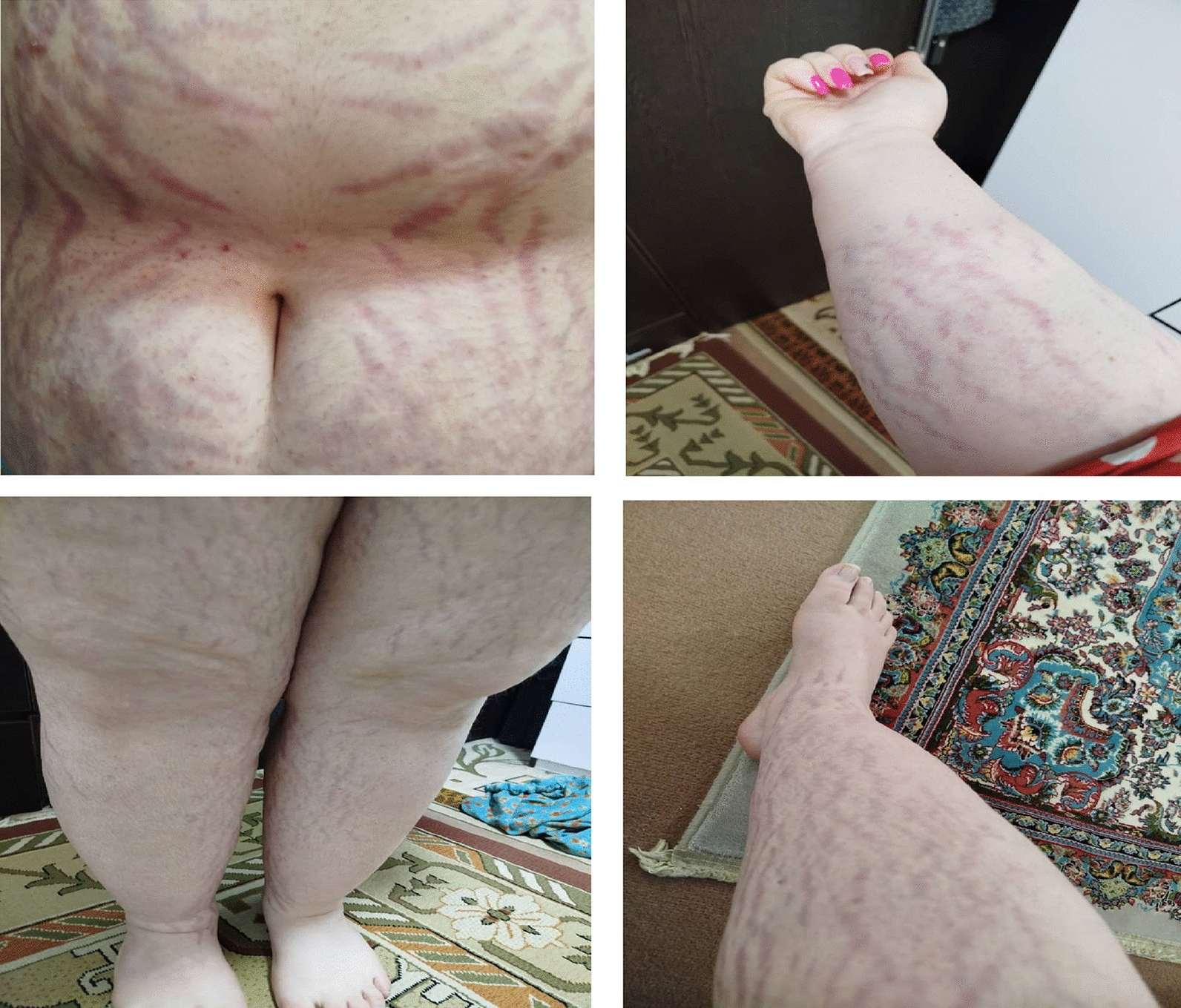
Clinical photograph showcasing the red–purplish striae on the patient’s abdomen, arms, and lower limbs

Figure 1 is a clinical photograph (with patient consent) or an illustration of the red–purple striae.

Upon admission, the patient’s history revealed prolonged use of betamethasone 0.1% 1 mg/mL nasal drops, administered at a daily dosage of 5 cc, in combination with oxymetazoline (a sympathomimetic nasal preparation) at a daily dosage of 1 cc, over approximately 12 months, to address nasal obstruction. Her symptoms began 6 months after starting the nasal drops. Further medication history revealed no other corticosteroid use. Notably, the patient had a past diagnosis of polycystic ovary (PCO) syndrome made on the basis of Rotterdam 2003 criteria (oligomenorrhea since menarche and clinically androgen excess) but did not undergo treatment or maintain laboratory records.

A detailed hormonal evaluation was undertaken. Morning plasma cortisol less than 0.05 µg/dL and adrenocorticotropic hormone (ACTH) less than 5 (10–56 pg/mL) measurements were abnormally low. Her 24-hour urine-free cortisol concentrations of 1.04 µg/24 h were significantly reduced, indicating suppression of the HPA axis secondary to prolonged exogenous corticosteroid exposure. All tests were repeated several times by endocrinologists during the time course of disease manifestations.

Table 1 summarizes the hormonal test results to clearly display the abnormalities.

**Table 1:** Hormonal and biochemical test results with reference values

Test	Result	Unit-reference value
Cortisol (AM)	*Less than 0.05*	5–25 μg/dL
ACTH (AM)	*Less than 5*	10–56 pg/mL
T4	*6.98*	5.1–14.1 μg/dL
T3	*0.85*	0.92–2.33 nmol/L
TSH	*1.83*	0.3–5.4 μIU/mL
LH	*12.77*	Follicular phase: 2.4–12.6 IU/L, ovulatory phase: 14.0–95.6 IU/L, luteal phase: 1.0–11.4 IU/L
FSH	*5.14*	Follicular phase: 3.9–12 IU/L, ovulatory phase: 6.3–24 IU/L, luteal phase: 1.5–7 IU/L
Prolactin	*26.32*	4.79–23.3 ng/mL (nonpregnant)
DHEA-S	*24.83*	148–407 μg/dL
17OH-progesterone	*1.87*	Follicular phase: 0.4–1.51 ng/mL 1.42–4.51 ng/mL
Aldosterone (upright)	*42.40*	3.7–43.2 ng/mL (upright) Follicular phase: 3.7–31 (supine)
Renin (upright)	*25.71*	5.3–99.1 μIU/mL (upright) 4.2–59 μIU/mL (supine)
Vitamin D (25 OH)	*25.71*	Deficiency: < 20 ng/mL Insufficiency: 20–30 ng/mL Sufficiency: 30–50 ng/mL Toxicity: > 100 ng/mL
Urine-free cortisol (24 h)	*1.04*	10–55 μg/day
Cortisol after dexamethasone	*0.05*	μcg/dL

ACTH: Adrenocorticotropic Hormone, TSH: Thyroid-Stimulating Hormone, LH: Luteinizing Hormone, FSH: Follicle-Stimulating Hormone, DHEA-S: Dehydroepiandrosterone Sulfate

Imaging studies before admission included a computed tomography (CT) scan of the adrenal glands, which showed that both adrenal glands were of normal size. However, a dynamic pituitary magnetic resonance imaging (MRI) revealed an 11 mm pituitary gland, despite there being no rationale for imaging studies in this scenario.

The patient was counseled extensively about the condition, and betamethasone nasal drops were discontinued immediately. Ear, nose, and throat (ENT) consultation revealed normal findings and the psychiatric team diagnosed her with major depressive disorder (MDD). She was discharged on 15 mg prednisolone with a structured tapering plan to allow for gradual recovery of adrenal function and to prevent acute adrenal insufficiency. Follow-up appointments were scheduled to monitor her clinical progress and re-evaluate her HPA axis recovery.

## Discussion

This case highlights the rare but significant occurrence of iatrogenic CS secondary to prolonged use of intranasal betamethasone. Although oral corticosteroids are well-known to cause HPA axis suppression, INS is generally considered safer owing to their localized effects and lowering systemic absorption side effects. However, the associated potential of systemic absorption in INS remains a concern [13]. As demonstrated in this case, prolonged use of potent formulations such as betamethasone can lead to significant systemic effects, particularly when administered inappropriately or at high doses.

Betamethasone nasal drops, although effective for treating nasal congestion and inflammation [14, 15], carry a potential risk of systemic absorption through the nasal mucosa. Factors, such as prolonged use [6, 16, 17], and high potency [18], can significantly increase systemic bioavailability. R. J. Perry *et al*. [19] in study of seven children highlights that even patients receiving doses within conventional safety ranges may exhibit varying sensitivity to glucocorticoids, leading to symptomatic adrenal suppression or glucocorticoid excess. Unlike newer corticosteroid compounds, such as fluticasone or mometasone, which undergo extensive first-pass metabolism in the liver, betamethasone exhibits minimal hepatic metabolism, contributing to its prolonged systemic activity [20, 21]. This pharmacokinetic profile underscores the need for careful regulation and monitoring of its use, even in ostensibly localized therapies.

The clinical manifestations in this patient, including central obesity, striae, hirsutism, and mood changes, were classic features of CS and guided the diagnostic process [22]. Scutelnicu *et al*. [23] reported a case of a patient in the second trimester of pregnancy who, owing to chronic sinusitis, underwent intranasal betamethasone spray therapy. The patient manifested extensive striae on the lower limbs, as well as edema in the legs, arms, and face, accompanied by a weight gain of 22 kg over 3 months. After switching the patient’s treatment to an alpha-1 adrenergic agonist spray, the condition was managed uneventfully without any symptoms of adrenal insufficiency.

Requesting imaging assessments, including a CT scan and MRI, as a first step further complicated the diagnostic process. This highlights a common diagnostic pitfall: the use of imaging as an initial approach can lead to the discovery of incidentalomas, which may misdirect clinical attention. Such findings risk overshadowing the primary etiology of the condition, potentially resulting in misdiagnosis or delayed treatment. This emphasizes the importance of prioritizing functional assessments over imaging in the early diagnostic workup to avoid unwarranted diagnostic confusion and ensure accurate identification of the underlying pathology.

Management involved the immediate cessation of betamethasone nasal drops and initiation of a structured tapering regimen with prednisolone to support adrenal recovery. The importance of stress-dose precautions during intercurrent illnesses was emphasized, alongside comprehensive patient education to prevent future misuse of corticosteroids. The gradual improvement in adrenal function during follow-up highlights the reversibility of glucocorticoid-induced adrenal suppression with appropriate intervention.

## Conclusion

This case underscores several critical lessons. First, it emphasizes the importance of heightened awareness among healthcare providers regarding the potential systemic effects of topical corticosteroids, particularly potent formulations such as betamethasone. Second, it highlights the need for thorough history-taking and detailed patient education to prevent corticosteroid misuse. This report contributes to the limited body of literature on iatrogenic CS from intranasal corticosteroids, particularly in adults. Documenting the clinical presentation, diagnostic challenges, and successful management of this case, provides valuable insights into preventing, recognizing, and treating similar cases. It serves as a reminder of the delicate balance between therapeutic benefit and potential harm in corticosteroid therapy and advocates for ongoing research to establish safer prescribing practices.

## Data Availability

The data analyzed and generated in this study can be accessed through the corresponding author upon reasonable request.

## Abbreviations

CS: Cushing’s syndrome
INS: Intranasal corticosteroids
HPA axis: Hypothalamic–pituitary–adrenal axis
BMI: Body mass index
FGS: Ferriman–Gallwey Score
PCO: Polycystic ovary
ACTH: Adrenocorticotropic hormone
CT: Computed tomography
MRI: Magnetic resonance imaging
ENT: Ear, nose, and throat
MDD: Major depressive disorder
